# Correlation between Human Embryo Morphokinetics Observed through Time-Lapse Incubator and Life Birth Rate

**DOI:** 10.3390/jpm14101045

**Published:** 2024-10-09

**Authors:** Laura Maghiar, Petronela Naghi, Ioana Alexandra Zaha, Mircea Sandor, Alin Bodog, Liliana Sachelarie, Georgiana Vieriu, Liana Stefan, Anca Huniadi, Loredana Liliana Hurjui

**Affiliations:** 1 Calla-Infertility Diagnostic and Treatment Center, Constantin A. Rosetti Street, 410103 Oradea, Romania; lauratodan@yahoo.com (L.M.); petronelanaghi@gmail.com (P.N.); drzahaioana@gmail.com (I.A.Z.); caita.georgiana@yahoo.com (G.V.); lianaantal@gmail.com (L.S.); ancahuniadi@gmail.com (A.H.); 2 Faculty of Medicine and Pharmacy, University of Oradea, 1st December Square 10, 410073 Oradea, Romania; drims75@yahoo.com; 3 Pelican Clinical Hospital, Corneliu Coposu Street 2, 410450 Oradea, Romania; 4 Department of Clinical Discipline, Apollonia University, 700511 Iasi, Romania; 5 Oradea County Hospital, Gheorghe Doja Street 65-67, 410169 Oradea, Romania; 6 Department of Medical Disciplines, Faculty of Dentistry, “Grigore T. Popa” University of Medicine and Pharmacy, 700115 Iasi, Romania; loredana.hurjui@umfiasi.ro

**Keywords:** time-lapse incubator, morphokinetics, embryo evaluation

## Abstract

(1) Background: Does the variation in sequential development times of embryos, observed through time-lapse monitoring, between the two study groups play a role in predicting pregnancy success? (2) Methods: The prospective double-arm study was to identify the morphokinetic parameters specific to embryos that were capable of implanting and were conducted on 89 embryos cultured in the Esco Miri time-lapse incubator, divided into two groups: Lot A, consisting of 57 embryos that successfully implanted and resulted in life birth rate (LBR), and Lot B (NLB), comprising 32 embryos that did not implant, leading to a negative beta-hCG outcome. (3) Results: Baseline characteristics, including female age, were not found to be statistically significant (*p* > 0.01). In contrast, there is a highly statistically significant difference concerning oocytes (*p* = 0.0029). Morphokinetic variables represented by sequential culture times were not statistically significant (*p* > 0.01) when comparing the two groups. However, the negative mean differences between these parameters suggest that the times for Lot A are better (shorter) than those for Lot B. While not statistically significant, these differences may still have practical significance. In the case of grading, the difference is considered to be extremely statistically significant (*p* < 0.01). (4) Conclusions: Although there are no statistically significant differences in sequential timings (*p* > 0.01) between the two groups, there are parameters indicating predictive potential for exploring pregnancy in embryo morphokinetics.

## 1. Introduction

The aim of all assisted reproductive techniques (ART) is to increase the pregnancy rate and, consequently, the birth rate. To achieve this, embryos with implantation potential are needed. Several methods for selecting embryos for transfer or vitrification are used, and these utilize morphological and morphokinetic aspects, as well as various OMICS such as metabolomics, proteomics, transcriptomics and genetic screening [1,2,3].

In vitro fertilization (IVF) and intracytoplasmic sperm injection (ICSI) are methods by which a woman’s eggs and a man’s sperm are combined outside the body to achieve fertilization. The embryos are then kept in an incubator and transferred to the woman’s uterus between the second and fifth days of development [2,3,4]. Typically, embryos are removed from the incubator for microscopic assessment of their quality and development stage. However, a Time-Lapse System (TLS) can capture images of embryos at frequent intervals, allowing for evaluation without removing them from the incubator. Additionally, TLS software (version 7.9) can assist embryologists in selecting the highest-quality embryo for transfer, thereby increasing the chances of a successful pregnancy [4].

To improve the pregnancy rate, the IVF laboratory monitors the internal environment of the laboratory—temperature, humidity, brightness, and the incubators’ internal environment—pH, temperature, CO_2_, O_2_, and N_2_ pressure; to maintain optimal conditions for embryo cultures [4]. However, when embryos are removed from the incubator to check their developmental stage, variations occur in the medium’s pH, temperature, and osmolarity. These variations variably affect, undetectably, the quality of the evolution of zygotes and embryos. To continuously monitor embryos in the internal environment of the incubator, a time-lapse incubator with permanent video recording of the embryos during their development up to the blastocyst stage has been developed [5]. Laboratories are required to update to new technologies available on the market continuously and to quickly adapt to the algorithms of artificial intelligence, which offer various prognostics regarding human gametes, fertilization rate, implantation rate, fragments, and cells excluded during the evolution of the embryo until the blastocyst stage [6], and to identify the morphokinetic parameters used in the selection/deselection of embryos, and how the morphokinetic parameters are monitored [7].

Models for selecting embryos based on morphological and morphokinetic characteristics use basic statistical methods, for example, logistic regression [8,9], while models for selecting embryos with OMICS use artificial intelligence with artificial neural networks [10,11]. Manual annotation by the embryologist is considered subjective and depends on the visual acuity of the observer, has many variables, and is not reproducible [12,13,14,15]. Artificial intelligence applies automatic annotation based on the morphological and morphokinetic characteristics of embryos: tpb (time to polar body), Tpn (time to pronuclei), juxtaposition of pronuclei, t2 (Multiple appearance of two cells), t3, t4, t5, t6, t7, t8, t9, TE thickness, embryo diameter, ICM area, and division speed [16].

Although assessment of the morphology of the embryos using static microscopic examination is considered to be subjective and time-lapse imaging is supposed to be an advanced technique, still there is a lack of data regarding the consistency of the measurements obtained through time-lapse parameters.

The application of morphokinetics, which captures the timing of embryonic developmental events and dynamic morphology through continuous time-lapse monitoring, adds a new layer to traditional morphology classification scores [12,13,14]. Using time-lapse imaging, Wong et al. identified three specific cell cycle events occurring before genomic activation, which were associated with successful blastocyst development [15]. Further reviews of morphokinetic research suggest that variations in the timing of these embryonic events could provide deeper insights into embryonic potential. Numerous retrospective studies have been conducted to explore how specific cell cycle kinetic parameters correlate with blastocyst formation and the likelihood of achieving pregnancy [7,12,15,16,17,18,19,20,21,22,23].

In a follow-up investigation, the same research group retrospectively analyzed pregnancy outcomes by comparing standard culture and traditional embryo grading to those obtained using the Embryoscope along with time-lapse parameters for embryo selection, observing higher pregnancy rates with the time-lapse system [9]. Additionally, a recent randomized controlled trial by Rubio et al. demonstrated that continuous time-lapse culture and selection significantly improved ongoing pregnancy rates compared to standard embryo culture, thereby corroborating the model initially introduced by Meseguer et al. [1,3,24,25,26,27].

Our study aims to identify which morphokinetic characteristics of the embryos cultured in the time-lapse incubator are positively associated with pregnancy. Various critical intermediate timings, including second polar body appearance, pronuclei appearance, syngamy, pronuclei disappearance, and multiple metotic divisions, could be different among embryos leading to pregnancy and embryos that do not implant; therefore, their identification could lead to better embryo selection.

## 2. Materials and Methods

This prospective double-arm study was conducted at the Calla—Infertility Diagnostic and Treatment Center, located in Oradea, Romania, over 2 years. The study received approval from the Ethics Committee, under reference number 670 on 11 November 2021.

Each embryo was followed from the time of fertilization to Day 5 or Day 6, when the decision was made to transfer or freeze the embryo.

### 2.1. Study Design and Population

**Figure 1:**
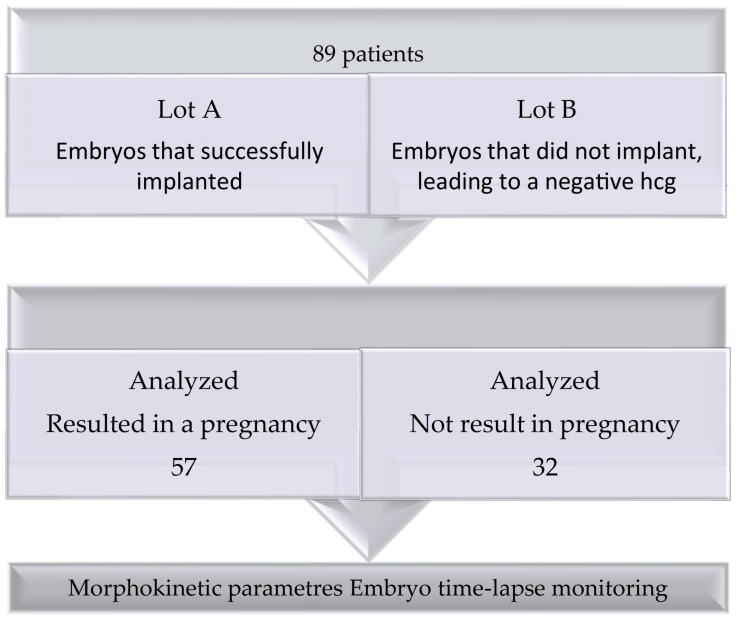
Workflow.

A total of 89 patients were enrolled in this study and were divided into two groups: Lot A included 57 patients who had embryos that implanted successfully, resulting in live births, and Lot B included 32 patients whose embryos did not implant, leading to a negative HCG. The difference between the two analyzed groups underlines the potential impact of morphokinetic characteristics on implantation success. The flowchart of the included patients is related in Figure 1.

Inclusion criteria were couples diagnosed with infertility, including male factor infertility, such as teratozoospermia and oligoteratozoospermia; and female factor infertility, such as tubal infertility, low ovarian reserve, and polycystic ovary syndrome (PCOS).

Exclusion criteria were embryos derived from donated oocytes and/or sperm, and patients with genetic conditions or medical contraindications to assisted reproductive technologies (ART).

### 2.2. Ovum Pick up Si ICSI (Intracytoplasmic Sperm Injection)

After aspiration of the follicles, the harvested oocytes were washed by the follicular fluid in G-MOPS medium (Vitrolife) (Denver, CO, Country: USA) and were incubated for 3 h in G-IVF medium (Vitrolife) in the Esco MIRI incubator, under conditions of 5% O_2_, 5.5% CO_2_. After incubation and equilibration of the oocytes in the G-IVF environment, they were stripped with Hyaluronidase Hyase (Vitrolife) and mechanical pipetting using stripping tips, 150, 135, 125 µm according to the Vitrolife manufacturer protocol.

After stripping the oocytes, ICSI was performed in drops of GMOPS (Vitrolife) medium, covered with OVOIL (Vitrolife) oil at 400× magnification, under a NIKON Eclipse Ti_2_ microscope. Metaphase II oocytes were injected, with spermatozoa from the fresh sample, processed by migration with the Zymot device, using the Spermactive culture medium (Gynemed Co., Rosedale, MD, USA), balanced overnight in a CO_2_ incubator. To slow down the motility of the spermatozoa, the ICSI-PVP medium was used (Fertipro, Beernem, Belgium).

### 2.3. Incubation and Culture Conditions

The injected oocytes were placed in culture dishes, Esco cultureCoin, for MIRI Time Lapse. Each culture vessel contains 14 central wells for embryo culture, plus washing wells, and captures high-resolution images of embryos at regular intervals, typically every 10–15 min. This system successfully selects embryos, providing information on their developmental patterns and potential for successful implantation.

Esco culture coin culture vessels are prepared 24 h in advance: in each well, for embryo culture, 24 microliters of G-TL medium (Vitrolife) are introduced, plus 23 microliters in the washing wells. Cover with 4 mL of OVOIL mineral oil (Vitrolife), and leave to equilibrate overnight, in a 5.5% CO_2_, 5% O_2_ incubator. Before loading the oocytes, check the culture vessel and remove the air bubbles under microscopic control. The injected oocytes are loaded very precisely in the center of the central well, for blastocyst culture, 5/6 days, under conditions of 5% O_2_ and 5.5% CO_2_.

### 2.4. Morphokinetic Parameters Analyzed in Embryo Time-Lapse Monitoring

Based on the digital images, the time of the parameters that precede fertilization and the biomarkers of fertilization are followed: the emission of the second polar body, t PB—the time of appearance of the second polar body; the appearance of the fertilization cone—changes in the cortex of the oocyte determined by the DNA of the sperm; the time of the appearance of the cytoplasm wave-cytoskeletal rearrangements of the cytoplasm of the oocyte, determined by the sperm and precedes the appearance of the male pronucleus, the time of the appearance of the female pronucleus, the juxtaposition of the pronuclei, the appearance of the cytoplasmic halo, the disappearance of the cytoplasmic halo, the rupture of the membranes of the female pronucleus, the rupture of the membranes of the male pronucleus, and the time of the first cleavage [12] of fertilization, is followed at 17–18 hpi; considered Day 2 at 42–44 hpi, Day 3 at 66–68 hpi, Day 4 at 90–92 hpi, Day 5 at 114–116 hpi, and Day 6 at 138–140 hpi (hpi-hours from insemination).

CC1 is the first cell cycle and describes the events that occur from the disappearance of pronuclei to the two-cell stage, CC1 = t2 − tPNf [13,14]. The time for DNA replication is the time when pronuclei appear t PN and pronuclei disappear—tPNf. Synchronization of divisions S1 = tPNf − Tpn.

CC2 is the second cell cycle and describes the events that take place starting from the zygote with two blastomeres, which divide and form two cells/blastomeres each, CC2 = t3 − t2.

S2 synchronization of the second cell cycle is defined as the transition from two blastomeres to four blastomeres, S2 = t4 − t3.

CC3, the third cell cycle, describes the events during which the embryo evolves from four blastomeres to eight blastomeres, CC3= t5 − t3. Synchronization S3, of the third cell cycle, begins with the division of the first blastomere out of the four, S3 = t8 − t5 [13,14]. Embryos were annotated at time intervals, corresponding to the number of cells, respectively: t5, t6, t7, t8.

Compaction time, tcomp, or morula time—Compaction can be early, starting on Day 3 of development, approximately 50 h, or late compaction at 105 h. The time-lapse recordings show that the compaction starts at one point of the embryo and extends throughout the embryo. During this time, the cells that are excluded from the mass of the embryo, certain fragments, are followed. Compaction can be complete when all cells are included in the embryo mass and incomplete when there are cells excluded from the embryo mass [16].

The early blastocyst time, teB, represents the time when the embryo begins cavitation with the formation of the blastocoel and the polarization of cells from the outside with the formation of the trophectoderm. It is important to observe the exact moment in the initiation of the cavity. The blastocyst time, t B, is defined as the time when the blastocell encompasses the entire embryo and the embryo grows in diameter.

### 2.5. Grading Embryos

Blastocyst formation varies between 31% and 65%, depending on the laboratory, the culture media, and the patient background [18].

The Gardner system was used to grade the embryos, the 1–6 scale was used for the degree of blastocoel expansion, and A, B, C grading was used for ICM and TE [19].

### 2.6. Embryo Transfer

The embryo transfer was carried out with a single embryo that had the highest implantation potential. Surplus embryos were vitrified, following the Kitazato protocol for vitrification. The beta HCG test was performed 7/8 days after the embryo-transfer procedure.

### 2.7. Statistical Analysis

Descriptive statistical analysis was performed using SPSS Statistics SPSS 26.0 (IBM SPSS Software, SPSS, Inc., Chicago, IL, USA). Data were presented as mean ± SD for continuous variables, and a samples *t*-test was used to determine variables.

## 3. Results

### 3.1. Baseline Characteristics of Groups

The average age of females in Lot A was 35.28 ± 5.13 and 37.09 ± 3.10 in Lot B, respectively. The number of oocytes were 14.48 ± 5.41 and 10.36 ± 3.51, per Table 1.

Baseline characteristics, including female age, were not found to be statistically significant (*p* > 0.01). This suggests that any observed differences related to female age may have occurred due to random chance rather than being indicative of a meaningful effect on the outcomes studied.

The difference between the number of oocytes in Lot A and Lot B is considered to be very statistically significant (*p* = 0.0029, *p* < 0.01).

### 3.2. Descriptive Statistics Lot A

Table 2 and Table 3 illustrate the characteristics of the morphokinetics time of the embryos for both groups. In Lot A we calculated the mean of the times (t PB…) of the embryos that achieved a live birth, and in Lot B we did the mean of the morphokinetic times that did not achieve a live birth. Descriptive statistics for Lot A and Lot B are shown in Table 2 and Table 3.

The morphokinetic variables represented by sequential culture times were not statistically significant (*p* > 0.01) when comparing the two groups, per Table 3. However, the negative mean differences between these parameters suggest that the times for Lot A are better (shorter) than those for Lot B. While not statistically significant, these differences may still have practical significance.

The mean of Lot A minus the mean of Lot B results in a negative value, which means that Lot A has, on average, a lower value or score compared to Lot B. In other words, Lot B tends to have a higher value of times, on average, than Lot A in the context of the compared data. That is, in Lot A the times are better than in Lot B.

In the case of grading, the difference is considered to be extremely statistically significant (*p* < 0.01), per Table 4. This indicates that the differences observed in grading between the two groups are highly unlikely to be due to random chance and are most likely meaningful in the context of the study.

## 4. Discussion

Many authors have tried to find certain differences between the morphokinetic parameters annotated in the time-lapse incubators in order to predict the future of the embryos [5,10] more accurately. Many studies have framed the embryos: embryos that were implanted versus embryos with implantation failure, euploid embryos versus aneuploidy embryos, or in mosaic. The literature led to contradictory and even paradoxical results, with some authors stating that there are differences between the morphokinetic parameters of euploid embryos vs. aneuploid embryos, and other authors stating that there is no difference [28].

Basile et al. establish a risk model for aneuploidy, in which embryos with a low risk of aneuploidy have the parameter = t5 − t2 up to 20.5 hpi, and CC3 between the limits of 11.7–18.2 hpi, and risk embryos that exceed the limits of the above parameters have a high level of aneuploidy [10].

Campbell et al. establish another risk model for aneuploidy, which uses blastulation start time, tsB, and blastocyst time, tB. In this risk model for aneuploidy, embryos with low risk of aneuploidy have tsB below 96.2 hpi and t B below 122.9 hpi; embryos with medium risk of aneuploidy have tsB greater than or equal to 96.02 hpi and tB below 122.9 hpi; and embryos with high risk of aneuploidy have t B greater than or equal to 122.9 hpi [6].

Jingye Zhang et al. establish that there are no statistically significant differences between embryos that led to live birth and those that did not implant, and after compaction, the only statistically significant difference between euploid and aneuploid embryos is that aneuploid embryo have a longer interval between blastocyst time and early blastocyst time [29].

An attempt was made to establish some reference limits of the respective times, limits that predict the implantation potential and the pregnancy potential of the embryos. Different algorithms have been developed and try to solve this multifactorial dilemma, in which the embryo and the endometrium are also involved, and the interface between the two biological structures. In addition, each equipment and each laboratory should establish its own reference limits, knowing that these culture characters depend on the variety of the culture media, the environmental conditions in the laboratory, the consumables used, and the personnel.

In Table 2, the statistical difference observed between embryos that implanted and those with fertilization failure is related to their grading, and AA-graded embryos for internal cell mass (ICM) and trophectoderm (TE) have superior morphokinetic characteristics, indicating a maximum implantation potential.

In Table 3, we can see that the only significant difference, or close to a significant difference at the level of the cc2 parameter, is the duration of the second cell cycle.

The moment of the second cell cycle, cc2, starts with the embryo that has two blastomeres, cc2 = t3 − t2, and in embryos that have implanted, it has an average of 8.83 h compared to embryos with live birth and no implantation, where we have the value of 9.15 h.

For the batches of embryos with implantation versus those with implantation failure, by analyzing the morphokinetic parameters recorded in the time-lapse system (t PB, t PN, t syngamy, t PNf, t2, t3, t4, t5, t6, t7, t8, tcomp, t e BL, t BL), we found no statistically significant differences.

These findings have broader implications in the field of in vitro fertilization (IVF), potentially improving IVF success rates by considering factors like oocyte quality and grading. Clinicians may refine practices based on this research, emphasizing the significance of both statistical and clinical relevance in interpreting results. Larger, well-designed studies are warranted for further insights in reproductive medicine.

This study has limitations, including a small sample size, potential differences between statistical and clinical significance, and the interpretation of negative results. Caution is advised when applying these findings, and further research with larger samples and controlled designs is needed to confirm and extend these insights.

Besides kinetic parameters, there are other events observed through time-lapse monitoring that can aid in embryo selection. The detection of anomalous events such as direct cleavage and reverse cleavage, previously not possible with conventional static microscopy, can serve as useful deselection criteria. Direct cleavage, first studied by Rubio et al., was associated with lower implantation rates [14]. These researchers found an implantation rate of only 1.2% in embryos that divided from one to three cells in less than 5 h (t3 − t2). Laboratories implementing new time-lapse technology often encounter the challenge of how to proceed with its use. This study limited its sample size to 6 months of data collection to allow for a prompt analysis, aiming to gain an understanding of the morphokinetic patterns within our laboratory environment.

This study suggests that the identified morphokinetic parameters can improve embryo selection in IVF clinics, thereby improving implantation success rates and live births.

The findings of the study can be used to design an eventually standardized embryo-selection protocol based on the morphokinetic parameters identified in the study to provide viability scores and automated selection recommendations, thus facilitating the decisions of embryologists in IVF clinics.

Traditional embryo-selection methods are based on different observations of embryologists, which can lead to variations and inconsistencies in assessment. The integration of time-lapse analysis into selection processes allows clinicians to reduce the subjectivity of embryo evaluation, thus optimizing treatment strategies. The morphokinetic parameters from time-lapse technology render objective and quantifiable data, allowing a more precise and uniform selection of embryos based on concrete criteria. Therefore, further research with larger sample sizes and more rigorously controlled study designs is essential to verify these preliminary insights and to extend our understanding of the predictive potential of these parameters for pregnancy outcomes.

## 5. Conclusions

There are no statistically significant differences in sequential timings (*p* > 0.01) between the two groups. For predicting live births, there are parameters that indicate the predictive potentials, like the number of oocytes and the blastocyst grading. To validate and expand upon these findings, further research with larger sample sizes and controlled study designs is necessary.

## Figures and Tables

**Table 1:** Characteristics for Lot A and Lot B.

Age	N	Mean	SD	Mean_A-B_	t	df	*p* Value
Lot A	57	35.28	5.13	−1.81	1.4780	52	0.1454
Lot B	32	37.09	3.10				
Number of Oocytes	N	Mean	SD	Mean_A-B_	t	df	*p* value
Lot A	57	14.48	5.41	−4.12	3.1305	51	0.0029
Lot B	32	10.36	3.51				

**Table 2:** Descriptive statistics for morphokinetics time characteristics Lot A.

Morphokinetics Time Characteristics—Lot A	Mean	SD
t PB—Second polar body extrusion	4.3372	1.6116
t PN—Pronuclear appearance	11.5094	2.4026
T Syngamy—Breaking the membranes between the two pronuclei and mixing the nucleoli	19.5025	2.5857
t PNF—Pronuclear fading	22.1416	2.2257
t2—Time of appearance of the first two blastomeres	25.1981	2.2278
t3—Time of appearance of the first three blastomeres	33.9938	4.4105
t4—Time of appearance of the first four blastomeres	40.0500	6.1358
cc2—Time of appearance of the second cell cycle	8.8312	3.7950
S2—Duration of the transition of an embryo from two cells to four cells	6.1716	4.7075
t5—Time of appearance of five blastomeres	47.3772	6.1715
t6—Time of appearance of six blastomeres	53.1647	6.8748
t7—Time of appearance of five blastomeres	59.7972	8.3123
t8—Time of appearance of eight blastomeres	67.0963	7.5768
CC3—Time of appearance of the third cellular cycle	12.9491	4.1861
S3—Transition time of an embryo from four cells to eight cells	19.7034	6.2572
tcomp—Appearance time of the compaction of the morula	86.4097	7.3542
tearly bl—Time of appearance of the early embryo	99.4828	6.8996
Tblast—Time of blastocyst appearance on Day 5/Day 6	111.2832	7.74905
Grading—AA-1; BB-0	3.00	2.13

t—time measured in hours since insemination (hpi).

**Table 3:** Descriptive statistics for morphokinetics time characteristics Lot B.

Morphokinetics Time Characteristics—Lot B	Mean	SD
t PB—Second polar body extrusion	5.1023	1.2662
t PN—Pronuclear appearance	11.8486	2.5572
T Syngamy—Breaking the membranes between the two pronuclei and mixing the nucleoli	18.7905	3.1515
t PNF—Pronuclear fading	22.2168	2.2157
t2—Time of appearance of the first two blastomeres	25.2932	3.0559
t3—Time of appearance of the first three blastomeres	34.9227	4.9124
t4—Time of appearance of the first four blastomeres	39.4741	7.3265
cc2—Time of appearance of the second cell cycle	9.1541	4.2000
S2—Duration of the transition of an embryo from two cells to four cells	4.4505	4.9721
t5—Time of appearance of five blastomeres	46.8627	6.5410
t6—Time of appearance of six blastomeres	52.1118	7.0194
t7—Time of appearance of five blastomeres	60.0068	9.0786
t8—Time of appearance of eight blastomeres	67.8768	9.2853
CC3—Time of appearance of the third cellular cycle	12.3268	3.5428
S3—Transition time of an embryo from four cells to eight cells	21.1605	5.8318
tcomp—Appearance time of the compaction of the morula	85.9336	8.9347
tearly bl—Time of appearance of the early embryo	100.7109	6.9412
Tblast—Time of blastocyst appearance on Day 5/Day 6	109.9364	8.1423
Grading	1.23	1.80

t—time measured in hours since insemination (hpi).

**Table 4:** Comparations for morphokinetics time characteristics between Lot A and Lot B.

Morphokinetics Time Characteristics	Mean_A-B_	SED	t	*p* Value
t PB—Second polar body extrusion	0.0675	5.384	0.0125	0.9900
t PN—Pronuclear appearance	−4.2793	5.776	0.7409	0.4605
T Syngamy—Breaking the membranes between the two pronuclei and mixing the nucleoli	−8.0299	5.225	1.5369	0.1273
t PNF—Pronuclear fading	0.0675	5.318	0.1487	0.8821
t2—Time of appearance of the first two blastomeres	−2.2802	4.546	0.5016	−2.2802
t3—Time of appearance of the first three blastomeres	0.7431	4.180	0.1778	0.8593
t4—Time of appearance of the first four blastomeres	1.6342	4.533	0.3605	0.7192
cc2—Time of appearance of the second cell cycle	−11.4764	5.920	1.938	0.0553
S2—Duration of the transition of an embryo from two cells to four cells	9.8241	6.447	1.5237	0.1307
t5—Time of appearance of five blastomeres	2.4940	3.824	0.6523	0.5157
t6—Time of appearance of six blastomeres	−7.3751	4.324	1.7057	0.0911
t7—Time of appearance of five blastomeres	7.8200	4.933	1.5857	0.1160
t8—Time of appearance of eight blastomeres	−1.4930	4.818	0.3099	0.7573
CC3—Time of appearance of the third cellular cycle	−2.8628	5.352	0.5349	0.5940
S3—Transition time of an embryo from four cells to eight cells	−2.8879	4.357	0.6628	0.5090
tcomp—Appearance time of the compaction of the morula	−0.9308	6.118	0.1521	0.8794
tearly bl—Time of appearance of the early embryo	5.0732	6.121	0.8288	0.4091
Tblast—Time of blastocyst appearance on Day 5/Day 6	14.598	15.580	0.9179	0.3608
Grading	2.22	0.509	4.3625	0.0001

## Data Availability

Data are contained within the article.
